# Paternalistic Leadership and Safety Participation of High-Speed Railway Drivers in China: The Mediating Role of Leader–Member Exchange

**DOI:** 10.3389/fpsyg.2021.591670

**Published:** 2021-07-26

**Authors:** Na Zhang, Shuzhen Liu, Bowen Pan, Ming Guo

**Affiliations:** ^1^School of Economics and Management, Beijing Jiaotong University, Beijing, China; ^2^Management College, Beijing Union University, Beijing, China; ^3^School of Finance, Central University of Finance and Economics, Beijing, China

**Keywords:** paternalistic leadership, leader-member exchange, safety participation, high-speed railway drivers, safety management

## Abstract

This research aimed to examine the effects of paternalistic leadership on the safety participation of high-speed railway drivers. Survey data were collected from 601 drivers in major Chinese rail companies. Structural equation modeling was conducted to analyze the influence of paternalistic leadership on safety participation *via* leader–member exchange (LMX). The results indicated that moral leadership directly promotes safety participation. Besides, benevolent leadership was positively associated with safety participation. Also, LMX partially mediates the positive relationship between benevolent leadership, moral leadership, and safety participation. Therefore, paternalistic leadership promotes the safety participation of high-speed railway drivers.

## Introduction

Safety is key in high-speed railway (HSR) operation since any major railway accident causes significant loss of life and property. HSR drivers are crucial in railway operation systems, since they significantly impact railway safety (Davey et al., 2008; Guo et al., 2016). Drivers have to comply with safety rules and operational procedures to enhance emergency care and safe operation (Guo et al., 2019; Ye et al., 2019). Although “military management” is used in Chinese railway companies to control unsafe employee behavior, their management stresses on safety compliance and economic penalties over the development of safety participation among drivers. They have failed to set up a long-term safety participation mechanism and identify management-related factors causing low safety participation among drivers.

Unlike safety compliance, safety participation is associated with extra-role or organizational citizenship behaviors beyond the job scope (Meng and Chan, 2020). These include voluntary and self-initiated behaviors that improve safety (Griffin and Hu, 2013; Jiang and Probst, 2016), such as proactively helping coworkers resolving safety issues, attending safety meetings (Neal and Griffin, 2006), participating in safety-related training (Cree and Kelloway, 1997; Neal et al., 2000; Huang et al., 2012), and voicing safety concerns to managers (Mullen et al., 2011). In recent years, several studies have shown that organizational and managerial factors are essential in safety participation, with some studies identifying the role of leadership style.

Leadership styles, such as transformational leadership (Hoffmeister et al., 2014; Smith et al., 2016; Shen et al., 2017), ethical leadership (Chughtai, 2015; Wang et al., 2020; Wen et al., 2021), and destructive leadership (Balwant, 2021) shape employee safety performance, especially in safety participation. Some leadership styles are based on cultural background and leadership theories applied in the Western culture, which are not always suitable for Asian organizations (Bass, 1997; Shahin and Wright, 2004). However, most studies focused on Western or universally applicable leadership styles, such as transformational and transactional leadership, failing to account for the cultural role in safety management practices. Besides, no substantial discussion has been conducted on the role of leadership in safety participation in Asia.

Paternalistic leadership is significantly associated with traditional Chinese culture and is common among organizations in East Asian countries. Paternalistic leadership is superior to Western leadership in Chinese organizations since it is based on the leadership gap. Empirical research demonstrated that paternalistic leadership could predict job attitudes and performance among employees (Cheng et al., 2004). However, its impact on employee safety behavior is unknown. Besides, the effect of paternalistic leadership on active safety management participation, benevolence, morality, and authoritarianism in shaping safety behavior is unknown. In this study, the relationship between paternalistic leadership and safety participation of HSR drivers was explored (China).

Leadership behavior is also determined through a certain intermediary mechanism, so, what is the “black box” of paternalistic leadership influencing safety participation? This study showed that leadership behavior affects the attitude, behavior, and performance of employees by influencing and shaping the special superior-subordinate relationship. Social exchange theory, a reciprocal mechanism, explains the effectiveness of leadership. Monetary or non-monetary benefits to employees improve their effectiveness (Hansen et al., 2013; Lagowska et al., 2019). The social exchange theory shows that paternalistic leadership influences perceptions of social exchange quality, further influencing employee behaviors, such as safety participation. However, leaders and employees have different communication channels, thus different interactions. Therefore, it is necessary to explore the effect of paternalistic leadership on employee safety participation *via* the social exchange mechanism. Besides, leader–member exchange (LMX) can act as a bridge and tie between paternalistic leadership and employee safety participation.

This study attempts to make three major contributions. First, this study explores the relationship between paternalistic leadership and employee safety participation in the background of traditional Chinese culture, which enriches and improves the research on leadership and safety participation. Second, the study uses LMX as the intermediary variable between paternalistic leadership and safety participation, which helps people better understand the relationship between them. Finally, we take drivers of the high-speed railway industry as a research sample, which expands the application scope of paternalistic leadership and attracts attention to paternalistic leadership in the railway industry. Therefore, this study provides further guidance on safety participation, particularly in Chinese organizations.

### Paternalistic Leadership

Paternalistic leadership style is common in Chinese culture, commonly referred to as “a style that combines strict discipline, authority, fatherly benevolence, and moral integrity couched in a personalistic atmosphere.” Therefore, paternalistic leadership comprises three dimensions: benevolence, morality, and authoritarianism. Benevolent leadership is a holistic care behavior and includes individualized consideration, understanding, and tolerance. Moral leadership involves superior personal qualities, self-discipline, and selflessness, making a leader respected and emulated. Authoritarian leadership is an absolute authority and control behavior to ensure unquestionable obedience. In return, subordinates show cognitive reactions, such as “considerate in return,” “identify and imitate,” and “hold in awe and submit,” i.e., “*shien*” (granting favors), “*shude*” (setting a moral example), and “*liwei*” (inspiring awe or fear).

Paternalistic leadership is based on Chinese culture and stems from Confucian ideology. Confucianism advocates for people-oriented, harmonious, and collective value culture, creating a benevolent, moral, and authoritarian leadership, thus affecting the construction role and psychological cognition of subordinates (Karakitapoglu-Aygün et al., 2020; Nazir et al., 2020).

### Paternalistic Leadership and Safety Participation

Safety participation refers to exclusive participation in safety duties, such as attending safety meetings, volunteering to improve the safety plan (Cree and Kelloway, 1997), helping coworkers figure out safety-related issues, and improving the safety environment (Curcuruto et al., 2015; Wei et al., 2016). As an organizational agent, a leader can guide employees to achieve their goals and improve safety performance (Zohar et al., 2014; Schopf et al., 2021). Some studies have reported on the relationship between leadership and occupational safety outcomes, such as safety climate, safety consciousness, and safety behaviors, in the construction, mining, manufacturing, and petrochemical industries, indicating the importance of leadership in organizational management (Hofmann et al., 2003; Zohar and Luria, 2010; Conchie, 2013; Kim and Jung, 2019). Currently, scholars have been establishing systemic constructs to explain the influence of leaders on safety outcomes. They note that it is leadership behavior, rather than traits of leaders, that determines the effectiveness of leadership. Besides, they have identified positive leadership styles that promote leadership effectiveness. O'Dea and Flin (2001) conducted a study on offshore installation managers (OIMs) in the oil industry of the United Kingdom and identified active participatory leadership behavior as a key factor motivating employee safety participation. Barling et al. (2002) indicated that transformational leadership trust could improve employee safety awareness, promote effective communication, and stimulate safety participation of employees. Lu and Yang (2010) pointed out that safety concerns and safety behaviors by leaders significantly impact employee safety compliance, further confirming that leadership behavior influences employee safety behavior. The research of Clarke (2013) provides empirical support for the causal relationship between transactional leadership and safety performance and confirms that transactional leadership could improve employee safety participation. Jiang and Probst (2016) explored the impact of transformational leadership and destructive leadership on safety participation of the United States public transport drivers and found that transformational leadership with high safety motivation has a more significant impact on employee safety participation than low safety motivation. There is additional evidence for the relationship between paternalistic leadership and safety communication, where paternalistic leadership predicted the safety communication of a cabin crew (Chen, 2017). Therefore, we propose that paternalistic leadership will affect the safety participation of HSR drivers.

Benevolent leadership indicates the comprehensive concern of leaders for personal lives and the welfare of subordinates, specifically in individual care and forgiveness. Subordinates appreciate and reciprocate care and concern according to the traditional Chinese Taoist culture (courtesy demands reciprocity) and modern social exchange theory. Social exchange includes instrumental exchange and emotional exchange (Befu, 1977; Cropanzano and Mitchell, 2005; Kim and Qu, 2020). When leaders care about the lives and work of subordinates, the subordinates are “thankful for gratitude.” Subordinates will internalize the goals of an organization, spend more time on work, and demonstrate more safety behaviors in order to reward the care and concern of leaders.

Moral leadership demands distinguishing between public and private life and setting a good example. Subordinates respect and identify leaders when they are disciplined and selfless. Moral leadership emphasizes personal moral integrity, similar to the “accessible charm” of transformational leadership (Shaw et al., 2020). According to social learning theory, most human behaviors are learned by observing and imitating the behavior of demonstrators. In the workplace, leadership is the main learning mechanism and simulation object of employees, which also applies to moral leadership. When the behavior of a leader meets the ethical expectations of a subordinate, this can have a subtle and positive impact on the employee. When leaders value moral beliefs, which include safety objectives, they tend to inspire the recognition and imitation of employees.

Authoritarian leadership involves absolute authority and control over subordinates, demanding absolute obedience from subordinates. Leaders and subordinates have an “asymmetric superior-inferior relation,” causing fear and caution, thus greatly reducing the identification of employees with the organization. Besides, authoritarian leadership is “negatively associated with subordinates' work attitudes, such as commitment and satisfaction with team leaders” (Karakitapoglu-Aygün et al., 2021). Given the cultural background of high-power distance in China, it is quite common that demands of employees remain unsatisfied, resulting in resistance and anti-productive behaviors to vent their resistance, which are not conducive to the safety behavior of HSR drivers and inhibits employee safety participation. In light of the above discussion, the following hypotheses can be stated:

Hypothesis 1: Paternalistic leadership is related to safety participation.

Hypothesis 1a: Benevolent paternalistic leadership is positively associated with safety participation.

Hypothesis 1b: Moral paternalistic leadership is positively related to safety participation.

Hypothesis 1c: Authoritarian paternalistic leadership is negatively associated with safety participation.

### Leader–Member Exchange as a Mediator

Leader–member exchange refers to the relationship quality between leaders and employees at the psychological or interpersonal level (Graen et al., 1986). Limited resources hinder a close relationship between leaders and all subordinates in an organization. Some organization members are recognized and trusted because of their characteristics or work performance, becoming the “in-group” employees, while the rest are “out-group” employees. The social exchange theory shows that the “in-group” are few trusted employees to whom the leader usually grants more care, support, resources, and opportunities while they also generate positive feedback and reciprocity motivations, showing a high degree of loyalty to the organization or leader (Tarkang et al., 2020; Kim et al., 2021). However, the out-group are other employees who only receive relevant support as per the employment contract. They believe that they do not have the responsibility or obligation to repay their leader, thus lacking enthusiasm and initiative. Paternalistic leadership does not treat all subordinates equally, similar to governance and the social exchange theory.

The impact of paternalistic leadership on the safety behavior of HSR drivers requires a certain mechanism with exchange quality of leadership. This study indicated that LMX mediates the relationship between paternalistic leadership style and employee safety participation. Specifically, the response of employees to the behavior of their leaders is based on social exchanges. The quality of the LMX relationship has been considered fundamental to the attitudes and behaviors of employees (Buengeler et al., 2021). Studies have confirmed that leaders influence the exchange relationship quality, further affecting the attitude and behavior of employees. The social exchange theory also shows that employees desire rewards and work hard, undertake safe activities, and exhibit high performance if they obtain work resources and psychological support from leaders. Luria and Yagil (2010) showed that transformational leadership, characterized by a high-quality relationship with subordinates, promotes high group-safety outcomes.

Benevolent leadership, such as paying attention to the teaching, care, concern, and compassion of subordinates, promotes a high-quality exchange relationship. High LMX motivates employees, making them committed to safety-related works. The social exchange theory shows that people will perceive an obligation to reciprocate when they receive good intended treatments in social interactions (Gini, 1997), for instance, through safety participation in an organization. Benevolent leadership promotes feedback and safety concerns from employees, thus improving safety participation. Moral leadership includes many excellent qualities, such as integrity and fairness (Adler, 1983; Li et al., 2012), and these qualities are often key factors contributing to high LMX. Furthermore, benevolent leaders treat all employees equally and fairly, showing care and respect, and having trust in employees, meeting the psychological needs of employees, and helping them form high LMX. In return, employees display more safety-oriented practices, such as actively participating in safety meetings, taking the initiative to resolve safety issues, and promoting safety programs in the workplace to ensure the safe and smooth operation of HSRs.

In contrast, the authority and absolute control of authoritarian leadership may hinder the establishment of LMX. An authoritarian leader tends to demand absolute obedience and punishes disobedient employees, resulting in employees being unable to freely choose their work behavior. Consequently, employees will feel uneasy and oppressed and ultimately engage in negative social exchanges. Low LMX makes employees unwilling to undertake extra tasks because they fear making mistakes and being punished, thus reducing safety participation. Thus, we suggest the following hypotheses:

Hypothesis 2: LMX mediates the relationship between paternalistic leadership and safety participation.

Hypothesis 2a: LMX mediates the relationship between benevolent leadership and safety participation.

Hypothesis 2b: LMX mediates the relationship between moral leadership and safety participation.

Hypothesis 2c: LMX mediates the relationship between authoritarian leadership and safety participation.

**Figure 1:**
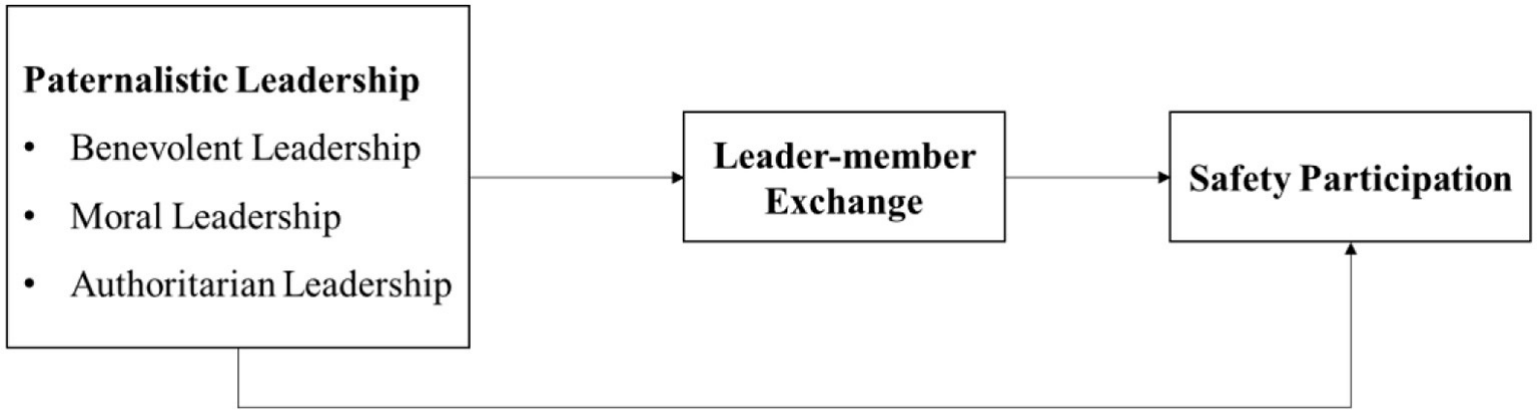
Conceptual model.

The theoretical model for the variables is shown in Figure 1.

## Methods

### Samples and Collection

This study used HSR drivers and their direct leaders (mentoring drivers or fleet captains). A total of 700 drivers were randomly selected between September 2019 and January 2020 from eight Chinese railway companies located in Beijing, Shanghai, Guangzhou, Hohhot, Zhengzhou, Wuhan, Taiyuan, and Kunming, China, with guarantee of anonymity and confidentiality. The participants were informed of the study aims and data collection procedures, and all had over 2 years of driving experience.

The data were collected in two stages. First, the participants were asked to report their perceptions of paternalistic leadership and LMX. All of them completed questionnaires independently and were not aware of specific results of each other. Each questionnaire included a researcher-assigned code to match the second-wave survey. The second wave of data collection consisted of surveying the direct leaders of the participants, who were required to complete the safety participation sections, i.e., to rate the safety participation of the participants in the survey. The researchers screened erroneous areas and removed invalid questionnaires to ensure data validity.

Of the 700 questionnaires that were distributed, 649 were returned, of which 601 were valid, and the effective response rate was 85.86%. All the participants were male with a mean age of 34.96 years (SD = 5.24 years). Besides, 52.6% of the participants had a junior college education (SD = 0.67). Most (60.4%) had not <10 years of train-driving experience (SD = 0.97 years). Most of the participants (61.2%) had spent over 3 years with their direct leaders (SD = 0.63 years). Detailed subject information is shown in Table 1. Most of the drivers and their superiors had a relationship for over a year. Thus, the evaluation of the relationship input and LMX is reliable.

**Table 1:** Demographic characteristics of respondents (*N* = 601).

**Demographics**	**Category**	**Frequency**	**Percentage**
Age	30 years old and below	159	26.50%
	31–35 years old	167	27.70%
	36–40 years old	162	26.90%
	Over 41 years old	113	18.90%
Education	Technical school and below	200	33.20%
	Junior college	316	52.60%
	University and graduate school	85	14.20%
Train-driving experience	5 years and below	238	39.60%
	6–10 years	167	27.80%
	11 years and over	196	32.60%
Technical title	Junior worker	2	0.30%
	Secondary worker	27	4.50%
	Senior worker	448	74.50%
	Technician	100	16.60%
	Senior technician	24	3.99%

### Measures

The specific measurement method for each variable is shown below. Relevant English scales were translated, back-translated, and culturally adjusted according to the cross-cultural adaptation program of the English psychometric scale recommended by Adler (1983) to avoid the language barrier. The relevant English scale was translated into Chinese and properly revised by combining it with information on the actual working conditions of the HSR drivers.

#### Paternalistic Leadership

The paternalistic leadership scale of Farh et al. (2000) comprising 26 items was used to determine paternalistic leadership. The drivers were asked to rate the paternalistic leadership of their direct supervisors (mentoring drivers or fleet captains) with 11 items on benevolent leadership, six items on moral leadership, and nine items on authoritarian leadership using a seven-point Likert scale [“strongly disagree” (1) to “strongly agree” (7)]. The sample items included: “He cares about me” (benevolent leadership), “He sets an example” (moral leadership), and “He asks me to completely obey his instructions” (authoritarian leadership). Acceptable Cronbach's alpha scale value was 0.89.

#### Safety Participation

The safety participation scale from Neal and Griffin (2006) with three items was used for safety participation analysis. The direct supervisor evaluated the safety participation behavior of the drivers using a seven-point Likert scale [“strongly disagree” (1) to “strongly agree” (7)], for instance, “I am actively involved in security-related meetings.” The acceptable Cronbach's alpha scale was 0.84.

#### Leader–Member Exchange

The scale of Liden and Maslyn (1998) was used to assess LMX. This scale evaluated the relationship between subordinates and supervisors in terms of emotion, loyalty, contribution, and respect. The scale has seven questions, and includes the following: “My supervisor helps me solve my work problems” and “My supervisor understands my work problems and needs.” The drivers responded to these items using a seven-point Likert scale [“strongly disagree” (1) to “strongly agree” (7)]. The acceptable Cronbach's alpha reliability was 0.74.

#### Control Variables

Demographic characteristics of employees are often used as control variables in paternalistic leadership analysis. The age, education, working time, and the time spent of the drivers with their direct supervisor were noted, similar to previous research (Wei et al., 2016). The age was coded as 1 = 30 or below, 2 = 31–35, 3 = 36–40, and 4 = 41 or above. Train-driving experience was coded as 1 = 5 years or below, 2 = 6–10 years, and 3 = 11 years or above. Education was coded as 1 = technical school or under, 2 = junior college, and 3 = university and graduate school and technical title was coded as 1 = junior worker, 2 = secondary worker, 3 = senior worker, 4 = technician, and 5 = senior technician.

### Analytical Strategy

The two-step approach of Gerbing and Anderson (1988) was used to assess the proposed research model. In the first step, confirmatory factor analysis was conducted to confirm the validity and reliability of the measurement model. The second step involved latent variable structural equation modeling (SEM) using the maximum likelihood algorithm in AMOS 23.

## Empirical Results

### Descriptive Statistics and Correlations

The descriptive statistics and a correlation matrix for the variables are shown in Table 2. Authoritarian leadership was negatively correlated with safety participation behavior of drivers (*r* = −0.14, *p* < 0.05), while benevolent leadership (*r* = 0.49, *p* < 0.01) and moral leadership (*r* = 0.54, *p* < 0.01) were positively correlated with safety participation, which provides initial support for the hypotheses.

**Table 2:** Descriptive statistics, correlations, and reliabilities for study variables.

**Variable**	**Mean**	**S.D**.	**1**	**2**	**3**	**4**
1. Benevolent leadership	4.71	1.33				
2. Moral leadership	5.38	1.23	0.74			
3.Authoritarian leadership	4.83	0.92	−0.01	−0.27		
4. LMX	4.32	1.00	0.59^**^	0.42^**^	−0.15^*^	
5. Safety participation	6.11	0.81	0.49^**^	0.54^**^	−0.14^*^	0.37^**^

* *p < 0.05*,

** *p < 0.01*.

### Reliability and Validity

A Cronbach's (1951) alpha coefficient and composite reliability (CR) were used to determine reliability. All the scales were highly reliable, close to or above 0.7 (Table 3) (Nunnally, 1987). The convergent validity and discriminant validity were also assessed. The average variance extracted (AVE) was between 0.49 and 0.59 for each construct, either equal to or higher than 0.5 (Fornell and Larcker, 1981; Bagozzi and Yi, 1988), indicating convergent validity.

**Table 3:** Reliability and validity for study variables.

**Variable**	**α**	**CR**	**AVE**
1. Benevolent leadership	0.94	0.94	0.59
2. Moral leadership	0.92	0.92	0.47
3.Authoritarian leadership	0.70	0.71	0.43
4. LMX	0.74	0.72	0.55
5. Safety participation	0.84	0.89	0.50

### Measurement Model

A confirmatory factor analysis (CFA) was performed to verify the measurement model *via* the maximum likelihood method. The model had acceptable fit indices [χ^2^ = 156.28, *p* < 0.001, χ^2^/df = 2.92, goodness-of-fit index (GFI) = 0.87, comparative fit index (CFI) = 0.91, Tucker–Lewis index (TLI) = 0.9, root mean square error of approximation (RMSEA) = 0.05], indicating a good fit to the data.

### Structural Model

**Figure 2:**
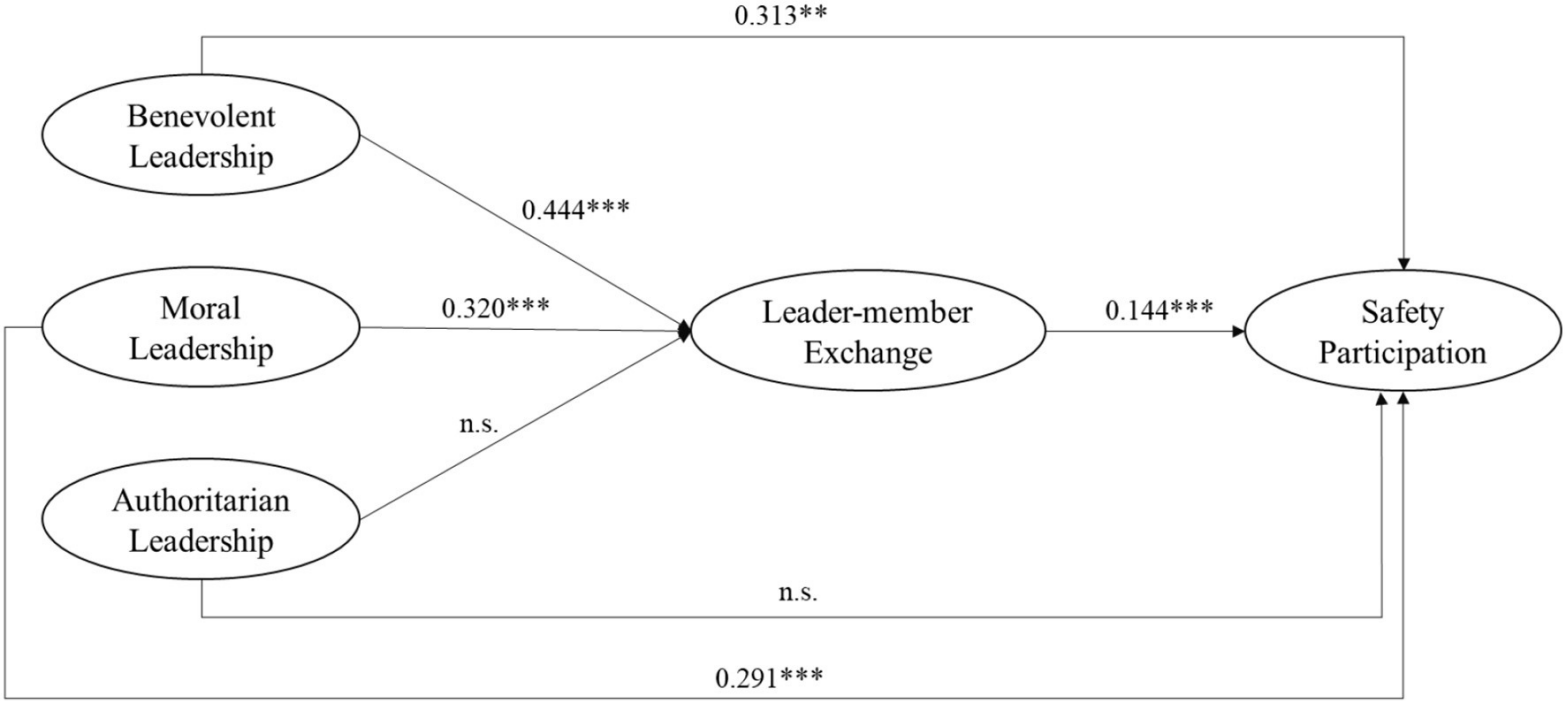
Structural model with LMX. Path coefficients are standardized. ***p < 0.001; **p < 0.01.

This study analyzed the structural models to confirm the proposed hypotheses using a maximum likelihood estimation method. The revised structural model had a good fit [χ^2^ (601) = 834.55; χ^2^/df = 2.74; CFI = 0.95; IFI = 0.95; GFI = 0.91, RMSEA = 0.05; SRMR = 0.07]. The standardized path coefficients for the final model are shown in Figure 2. Benevolent leadership had a positive effect on safety participation (β = 0.313, *p* < 0.001), supporting Hypothesis 1a. Moral leadership was also positively related to safety participation (β = 0.291, *p* < 0.001), supporting Hypothesis 1b. However, authoritarian leadership had no effect on safety participation (β = 0.04, *p* > 0.05), failing to support Hypothesis 1c.

Bootstrap analyses were also performed to examine the indirect effect of paternalistic leadership on safety participation *via* LMX (Shrout and Bolger, 2002). A total of 5,000 bootstrap samples were created, and a bias-corrected 95% confidence interval was used, following the Mallinckrodt et al. (2006) recommendations. The analytical results are shown in Table 4. Benevolent leadership had a significant mean indirect (unstandardized) effect of 0.263 and a bias-corrected 95% confidence interval of between 0.018 and 0.289, which did not include zero; thus, the indirect effect was statistically significant. Similarly, moral leadership had a significant mean indirect (unstandardized) effect of 0.322 and a bias-corrected 95% confidence interval of between 0.115 and 0.217, which did not include zero; thus, the indirect effect was statistically significant.

**Table 4:** Bootstrapping mediation testing results.

**Pathway**	**β**	**95% confidence interval**	
		**Lower**	**Upper**
Benevolent Leadership → Leader-member Exchange → Safety Participation	0.263	0.018	0.289
Moral Leadership → Leader-member Exchange → Safety Participation	0.322	0.115	0.217
Authoritarian Leadership → Leader-member Exchange → Safety Participation	0.026	−0.152	0.119

Therefore, LMX did mediate the effect of benevolent leadership and moral leadership on safety participation. However, bias-corrected confidence intervals of authoritarian leadership had zero, which does not support the hypothesized relationships. Therefore, the mediation model was partly confirmed, thus supporting Hypothesis 2a and 2b.

## Discussion

This study explored the relationship between paternalistic leadership and the safety participation of HSR drivers based on the social exchange theory. The mediating role of LMX in the relationship between paternalistic leadership and safety participation was also investigated. The results show that benevolent leadership and moral leadership, the two dimensions of paternalistic leadership, have a significant positive effect on the safety participation of high-speed rail drivers, while authoritarian leadership has no significant effect on the safety participation.

First of all, when leaders show kindness, high-speed rail employees will feel that the potential cost of safety participation is very low, and the expected risk and loss are small. As a reward for leaders' care and consideration, they will show a high degree of safety participation behavior. Therefore, benevolent leadership positively impacted the safety participation of the HSR drivers, consistent with Chan and Mak (2012). Second, we also found that the moral dimension of paternalistic leadership also promoted the safety participation of the HSR drivers, consistent with Gu et al. (2015). Employers learn from morally upright managers, and managers can fairly evaluate the behavior of employees. Employees are more likely to participate in safety activities, such as putting forward opinions or suggestions conducive for workplace safety when they trust the conduct of their leader. Finally, the study found that authoritarian leadership did not affect safety participation. This discovery is different from that of Chen (2017) regarding the influences of authority conduct on safety behavior of employees. However, Cheng et al. (2004) and Zhang et al. (2011) indicated that authoritarian leadership is not associated with employee performance, consistent with this study, since environmental changes influence parental leadership. Authoritarian leadership has been increasingly viewed as rational and instrumental. Authoritarian leadership has transformed from strict control of individual employees to control of work tasks and procedures. In contrast, subordinates have changed from absolute authority obedience to following the institutional norms established by the leadership. Therefore, the negative impact of authoritarian leadership was significantly reduced in this study.

Furthermore, this study also found that LMX mediates benevolent leadership, moral leadership, and safety participation, consistent with Lin et al. (2018) and Nazir et al. (2020) studies. The “Benevolence” and “Virtue” performance of the superior leader in daily work promotes a positive relationship with employees, i.e., LMX, reducing the leadership gap between managers and employees. Employees, as “insiders” of managers, then get involved in more activities that promote the safety of the organization. Therefore, as a bridge, LMX plays an intermediary role between benevolent leadership, moral leadership, and employee safety participation. However, this study also showed that LMX does not mediate authoritarian leadership and safety participation possibly because of the characteristics of employees. Most employees in modern enterprises have diversified values, strong self-awareness, a high level of knowledge, and no understanding of authority and traditional consciousness. Tang and Naumann (2015) also found that the Chinese no longer have a positive attitude toward authority obedience because of the young generation and social modernization, greatly weakening the social and cultural foundation of authoritarian leadership.

### Theoretical Implication

This research has several theoretical contributions. First, it enriches and improves the research on leadership and safety participation. Previous studies have confirmed the effect of differentiated leadership style on employee safety behavior. However, most studies are focused on transformational leadership, transactional leadership, and service leadership based on Western culture (Bian et al., 2019; Kim and Jung, 2019; Schopf et al., 2021), with few on paternalistic leadership based on Chinese traditional cultural values and ubiquitous in Chinese enterprises (Tian and Sanchez, 2017). This study explored the relationship between paternalistic leadership and employee safety participation based on traditional Chinese culture, greatly enriching and improving the relevant research on the above fields.

Second, this study reveals the influence mechanism of paternalistic leadership on safety participation based on the social exchange theory. Previous studies have shown that leaders influence subordinates through job stress, safety climate, and safety motivation (Kim and Jung, 2019; Xue et al., 2020; Basahel, 2021). In contrast, this study found that paternalistic leadership affects the safety participation of employees through employee–leader relationships. Therefore, paternalistic leadership directly affects safety participation and indirectly affects safety participation through LMX (social exchange mechanism).

Finally, this study broadens the scope of paternalistic leadership style application in the railway industry. Paternalistic leadership was first proposed by Redding (1996) and became common in Chinese companies after 20 years. Scholars have studied the influence of paternalistic leadership on psychology, attitude, and behavior of employees in military, manufacturing, high-tech enterprises, and other industries (Cheng et al., 2014; Chou et al., 2015; Huang and Lin, 2020), raising some important guidance and suggestions. The railway industry is also a typical enterprise with both enlightenment and power. This study takes drivers from the HSR industry as the research sample and shows that paternalistic leadership does have an important impact on safety participation, thus attracting the attention of scholars in the railway industry.

### Practical Implications

Studies have shown that over 90% of safety-related accidents are caused by unsafe human behavior. Therefore, it is necessary to promote the safe production of employees in safety management research. This study has several practical implications for HSR safety management. On one hand, the effect of paternalistic leadership on attitudes and behaviors of HSR drivers should be considered. For instance, universal paternalistic leadership substantially impacts the behavior of individuals in Asia. Therefore, organizations should select and train individuals who are benevolent and moral. Besides, managers should adopt a benevolence and morality style, demonstrating more “*shien*” (favor granting) and “*shude*” (setting an example) behaviors to create high-quality relationships, thus improving safety participation. Managers should not adopt an authoritarian leadership style, since it does not affect safety participation. Instead, they should prevent autocratic dictatorship.

This study also shows the importance of a positive relationship between managers and employees, for instance, the indirect effect of LMX on the safety participation of drivers. LMX has a certain practical guidance value. China values interpersonal relationships (Guanxi), where informal relationships help subordinates on formal organizational constraints (Lovett et al., 1999; Lin, 2011). Therefore, a manager should establish a high LMX relationship with subordinates, such as compassion, emotional care, rewards, and praise. The more active the interaction between the leader and driver, the higher the satisfaction of the driver with the work, work environment, and organizational environment, thus promoting safety participation.

### Limitations and Future Directions

This study also has several limitations. First, it used self-reported data. Although the data from the leaders and drivers were collected separately, data collected simultaneously can cause a causal inference challenge. Second, only LMX was selected as a mediating variable without considering the boundary conditions of the organization. Future research should examine other variables, such as safety climate (Zohar, 1980; Brown and Holmes, 1986; Silla and Gamero, 2018; Zhang et al., 2018) and other organizational-level or individual-level factors. Third, future studies should explore the impact of paternalistic leadership on other dimensions of safety behaviors, such as safety compliance, and compare it with the effect on safety participation to further clarify the relationship between paternalistic leadership and safety behavior. Fourth, the effect of paternalistic leadership on safety participation should be compared with that of other leadership styles, such as transformational and transactional leadership, to guide organizational safety management better. Fifth, this study focused on paternalistic leadership and safety participation in HSR, a more hierarchical culture than most industries. Future studies should examine the relationship between paternalistic leadership and safety participation in other industries.

## Conclusions

This study explored the impact of paternalistic leadership on the safety participation of HSR drivers based on the Chinese culture. Benevolent leadership and moral leadership positively influenced safety participation. It is also confirmed that LMX mediates paternalistic leadership and safety participation of HSR drivers. Benevolent leadership has high LMX, which positively affects safety participation. However, authoritarian leadership had no impact on safety participation and LMX. This research provides new insights into the use of the paternalistic leadership style in organizations to promote safety participation.

## Data Availability Statement

The raw data supporting the conclusions of this article will be made available by the authors, without undue reservation.

## Author Contributions

NZ and BP contributed to the idea and wrote the full manuscript. SL collected the data and run the data. MG revised the full manuscript and proposed improvements. All authors contributed to the article and approved the submitted version.

## Conflict of Interest

The authors declare that the research was conducted in the absence of any commercial or financial relationships that could be construed as a potential conflict of interest.

## Publisher's Note

All claims expressed in this article are solely those of the authors and do not necessarily represent those of their affiliated organizations, or those of the publisher, the editors and the reviewers. Any product that may be evaluated in this article, or claim that may be made by its manufacturer, is not guaranteed or endorsed by the publisher.
